# The Impact of Gender, Training Year, Medical Specialty and the COVID‐19 Pandemic on Resident Wellness

**DOI:** 10.1111/tct.70102

**Published:** 2025-06-08

**Authors:** Sara Beachy, Jessica Luzier, Chantel Weisenmuller, Kathleen Bors, Mary Ann Maurer, Amna Anees, Tiffany Lasky, Lisa Calderwood

**Affiliations:** ^1^ Department of Family and Community Medicine Thomas Jefferson University Hospital System Philadelphia Pennsylvania USA; ^2^ Department of Behavioral Medicine and Psychiatry Charleston Area Medical Center Charleston West Virginia USA; ^3^ WVU School of Medicine‐Charleston Division Charleston West Virginia USA; ^4^ Disordered Eating Center of Charleston Charleston West Virginia USA; ^5^ Department of Family Medicine Charleston Area Medical Center Charleston West Virginia USA; ^6^ Family Medicine Residency Program Charleston West Virginia USA; ^7^ Internal Medicine Residency Program Charleston West Virginia USA; ^8^ Department of Internal Medicine Charleston Area Medical Center Charleston West Virginia USA; ^9^ Trauma Surgical Critical Care and Acute Care Surgery, Department of Surgery Texas Tech University Health Sciences Center El Paso El Paso Texas USA; ^10^ Center for Health Services and Outcomes Research, Institute for Academic Medicine Charleston Area Medical Center Charleston West Virginia USA

**Keywords:** COVID‐19, gender, graduate medical education, medical, psychological well‐being, residency

## Abstract

**Introduction:**

Data are limited as to which factors most strongly impact medical trainees' well‐being, and few studies have assessed trainee's wellness at multiple time points during the COVID‐19 pandemic. Additionally, much research has not taken an intersectional approach to understanding the ways in which different facets of a resident's identity impact their wellness. Thus, more person‐centred, probability‐driven statistical approaches that can produce distinct heterogenous groups of individuals are warranted to understand the way in which different identities are associated with resident wellness scores.

**Methods:**

A latent profile analysis was conducted using institutional data from resident surveys administered from 2019 to 2022. We had a 78.8% response rates for a total of 1101 surveys completed and 1033 retained for data analysis (i.e., 662 medical residents, 204 surgical residents, 48 pharmacy residents and 119 residents who did not specify practice type).

**Results:**

Broadly, findings indicated that having certain identities (i.e., being female, being in a medical or pharmacy programme and length of time in programme) resulted in lower resident wellness scores. However, the degree to which these scores were impacted appeared to be dependent on the intersection of the residents' identities with COVID‐19 time point being moderately influential (e.g., advanced medical/pharmacy residents scored lower in latter part of COVID‐19; PGY1 medical residents scored lower regardless of COVID‐19 time point).

**Conclusions:**

Results from this study encourage data‐driven approaches to wellness initiatives that are tailored by gender, programme type and year of programme.

## 1 Introduction

Medical accrediting bodies include trainee wellness as a core competency in programming while striving to advance and improve the quality of education. Whereas many factors influence resident wellness, few studies examine resident wellness from an intersectional lens; however, programmes often implement broad institutional‐level wellness programming without consideration of nuances in residents' experiences. To complicate scholarship further, wellness and well‐being are frequently used interchangeably, which has led to a lack of theoretically grounded research and wellness programmes. The authors of a recent review proposed that wellness should include mental, social and physical health in addition to well‐being, which includes deriving meaning from life and work [1]. Therefore, wellness will be used in this manuscript as an umbrella term for well‐being.

Research demonstrates that resident identities may impact wellness. Female residents report higher levels of perceived stress, burnout and gender‐based discrimination and lower life security and overall wellness as compared with their male peers [2, 3, 4, 5]. Generally, residents who are farther along in training are more likely to report feeling their work is meaningful, greater life security and more institutional support than residents earlier in their training [3]. Unfortunately, there is more limited research to delineate differences in resident wellness across specialty areas and throughout the COVID‐19 pandemic. Broadly, COVID‐19 has been associated with increased distress, depression, anxiety and burnout among physicians, but little is known about its impact on resident wellness [6, 7, 8]. However, studies have found an initial spike in residents' concerns for their own wellness early in COVID‐19 with hospital‐based residents experiencing increased loneliness when compared to surgical residents [9, 10]. These findings suggest that COVID‐19 influenced resident wellness but that the pandemic's influence may depend on resident factors.

### 1.1 Present Study

The present study analysed factors that contribute to resident wellness using surveys administered from 2019 to 2022. As the scholarship is mixed on which factors influence wellness, we employed an intersectionality lens to help identify unique patterns in the data. Intersectionality was first introduced by Kimberlé Crenshaw and can be useful to identify the ways intersecting identities influence a person's lived experiences [11]. We conceptualised programme specialty, gender and year in programme as identities that influence the amount of power residents have in their respective institution and department and hypothesised that wellness scores would be lower in female and residents earlier in training.

## 2 Methods

### 2.1 Settings and Participants

Residents across all departments in a university‐affiliated hospital in the United States located in Appalachia were administered surveys between May 2019 and December 2022 by our institution's GME Office in cooperation with Wayne State University. Retrospective use of the data was approved by the hospital's Institutional Review Board (IRB) for the Protection of Human Subjects. We received 1101 completed surveys. Sixty‐three surveys were excluded as the individuals indicated they were not residents or respondents did not identify a gender. We chose to exclude these surveys because a response other than male or female could indicate (a) a non‐binary or gender non‐conforming identity, (b) the desire to remain anonymous in certain programmes or (c) missing data. Of note, we recognise the differences between gender and biological sex; however, as this was a retrospective study, we could not control for the way gender was asked in the demographics survey.

### 2.2 Outcomes

#### 2.2.1 Resident Wellness Scale

The Wayne State Resident Wellness Scale (RWS) is used by the Accreditation Council for Graduate Medical Education [3, 12]. This 10‐item, self‐report measure uses a 5‐point Likert scale to capture residents' experiences over a 3‐week period [3]. The RWS is based on Ryff and Keyes' theory of wellness and provides an overall score and four subscale scores: meaningful work/ability, life security, institutional support and social support/personal growth [3, 13]. We previously conducted a confirmatory factor analysis on the RWS using data from this same institute [14]. We found a factor structure similar to the 2‐factor structure identified by Appelbaum et al. sans item 3 [14, 15]. Therefore, two factors labelled as meaning (i.e., items 1, 2, 4, 5, 6 and 10) and human connections (i.e., items 7, 8 and 9) were used in the analysis. Reliability statistics for this study were acceptable for the total score (α = 0.89) and subscales (α = 0.74–0.87).

#### 2.2.2 Programme Type

Residency programmes were categorised as medical (i.e., paediatrics, behavioural medicine/psychiatry, emergency medicine, family medicine, internal medicine and cardiology), surgical (i.e., obstetrics/gynaecology, general surgery, vascular surgery and urology), pharmacy and unspecified.

#### 2.2.3 COVID‐19 Timeframe

Responses were categorised into four time periods based on survey completion: pre‐COVID‐19, prior to the first detected US case of COVID‐19; early COVID‐19, first US case until the first vaccine received emergency approval (1/30/2020–12/10/2020); Mid I COVID‐19, after the first vaccine was approved until omicron (12/11/2020–12/1/2021); and Mid II COVID‐19 (12/2/2021 to end of study).

#### 2.2.4 Other Demographics

Residents were coded as male (0) or female (1). Residents indicated their year (i.e., PGY1–PGY5).

### 2.3 Statistical Analyses

#### 2.3.1 Latent Profile Analysis

Mplus v. 8.9 was utilised for the latent profile analysis (LPA), whereas SPSS v. 29 was utilised for descriptive statistics. LPA is a person‐centred, probability‐based statistical analysis that groups participants together based on pattern of responses to items [16]. This analysis has been used to analyse intersecting identities and experiences on outcomes, thereby assessing intersectionality [17]. LPA assumes that people who score similarly on measures (e.g., wellness) have similar profiles (e.g., gender, year of residency, residency subtype and environmental factors—COVID time points) and groups people by calculating the probability for each person within each group [18]. In this analysis, an intersectionality lens would suggest that residents with differing levels of social power based on intersecting identities would have differing levels of wellness. Per best practices, LPA is iterative where multiple models with varying numbers of profiles are compared to one another. Multiple indices must be used to identify which model is the best fit for the data [16].

Fit indices (i.e., Akaike Information Criterion [AIC] and sample‐sized adjusted Bayesian Information Criterion [SABIC]), entropy, average latent profile probabilities, class size proportion and the bootstrapped likelihood ratio test were all simultaneously evaluated for each model [16]. The AIC and SABIC are information criteria fit indices that consider sample size and model complexity to help identify the ideal number of classes (i.e., highest quality model) where values closer to 0 indicate better fitting models [16, 19]. The parametric bootstrapped likelihood ratio test (BLRT) was evaluated with a significant result, indicating a meaningful difference between two solutions [16]. Entropy and diagonal probability values greater than 0.80 and 0.70, respectfully, indicated that individuals are classified appropriately into their respective class, which are well separated and well defined [16, 20]. Classes should be no less than 5%–8% of the sample and should be theoretically driven [20]. Conceptually, we anticipated smaller class sizes given the gender disparity in certain specialties. For example, female residents comprise only 18% of orthopaedic surgery residents [21]. Furthermore, 73% of these residents are European‐American, whereas number of Black, Latinx and Indigenous residents ranges between 0.2% and 7.2% [21]. Although we did not have access to racial makeup, we anticipated small classes based on shared gender and racial experiences related to power and positionality.

## 3 Results

### 3.1 Sample Demographics

Twenty‐eight per cent of the surveys (*n* = 294) were administered pre‐COVID‐19, 25% completed during early COVID‐19, 25% completed at COVID‐19 Mid I, and 22% completed at COVID‐19 Mid II. Most residents (*n* = 366) indicated that they were PGY1 (35%). Approximately 55% of respondents indicated they were male. Most residents were in medical (*n* = 662, 64%) and surgical programmes (*n* = 204, 20%). The remainder did not specify a programme (12%) or were in pharmacy residency (5%).

### 3.2 Latent Profile Modelling

**FIGURE 1:**
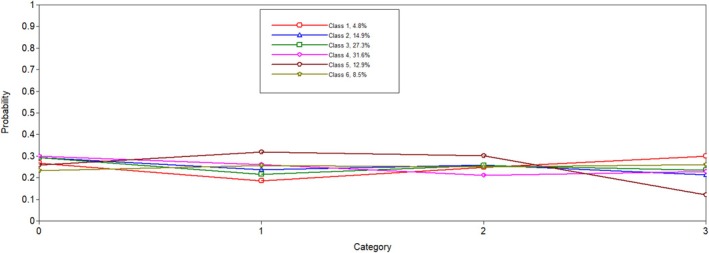
COVID‐19 time point.

**FIGURE 2:**
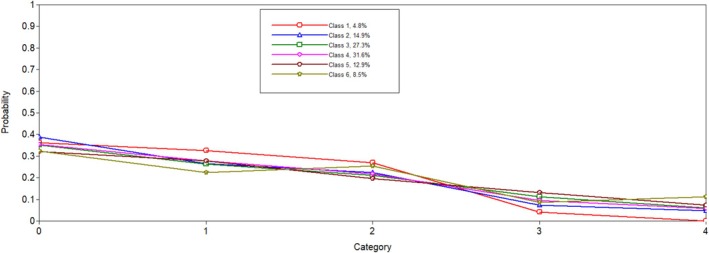
PGY.

**FIGURE 3:**
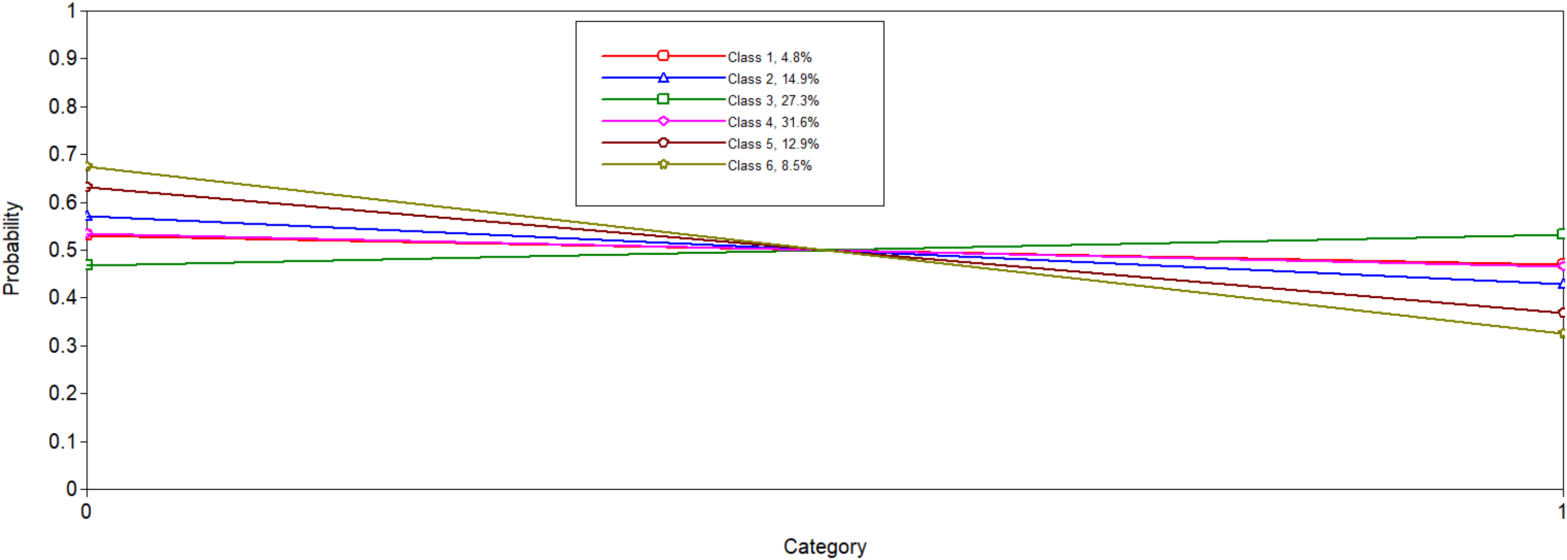
Biological sex.

**FIGURE 4:**
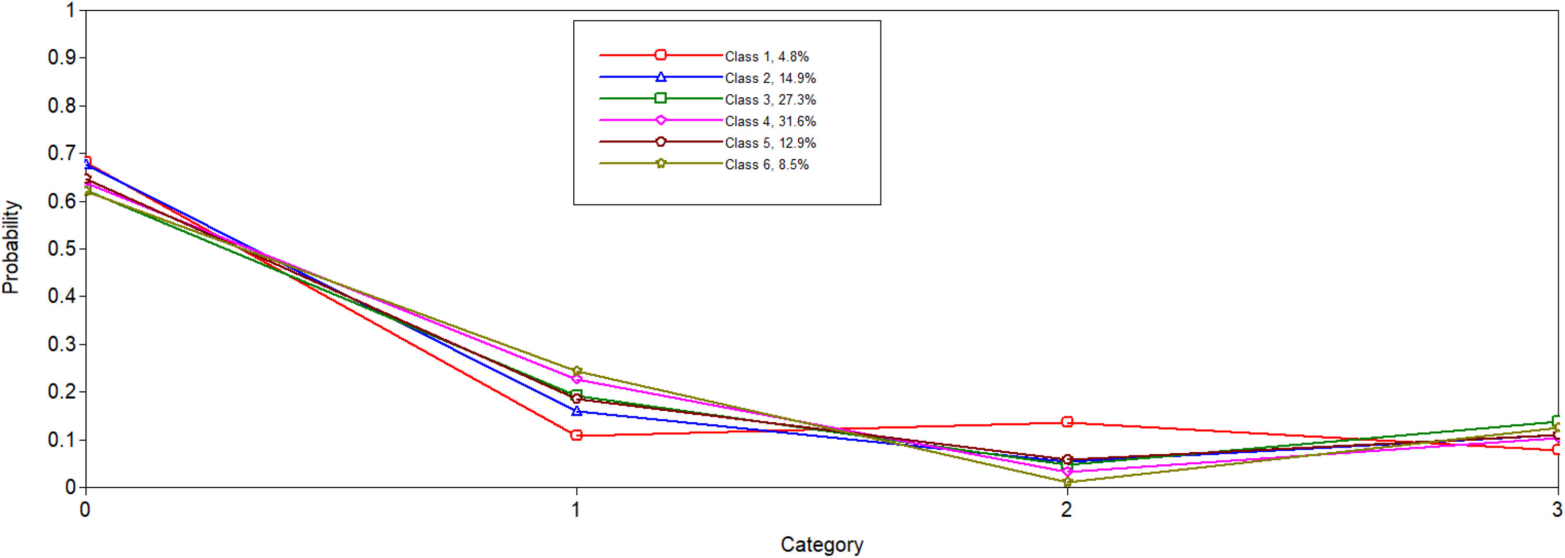
Programme type.

Six latent profiles analyses were conducted and compared to one another to determine the best solution. Notably, when the five and six latent profile analyses were conducted, a message on Mplus indicated that at least one logit threshold approached a 0 or 1, indicating a perfect prediction. Results were reviewed to ensure plausibility, and only one extreme logit threshold was identified. In both solutions, the probability of PGY5 residents in Latent Class 1 was 0. We determined this result was plausible given the low probability of individuals in this class being in surgical programmes, which are longer in length. When examining all fit indicators (i.e., entropy, AIC, SABIC, size of each class and average latent class probabilities; see Table 1), the six‐class model was favoured. The BLRT test for five classes versus six classes was significant (*p* < 0.001), which indicated that the six‐class solution fit better than the five‐class solution. The results of the latent class analysis are presented in Table 2. Additionally, Figures 1, 2, 3, 4 depict probabilities of each variable input (i.e., COVID time point, PGY, biological sex and programme type) by latent class.

**TABLE 1:** Goodness‐of‐fit indices.

Latent class	AIC	SABIC	ENTROPY
2	14878.16	14934.61	0.79
3	14176.68	14259.59	0.86
4	13599.15	13708.52	0.90
5	13343.19	13479.02	0.90
6	13186.18	13348.47	0.89

Abbreviations: AIC = Akaike Information Criterion, BIC = Bayesian Information Criterion, SABIC = sample‐sized adjusted Bayesian Information Criterion.

**TABLE 2:** Results from the latent class analysis with six classes present.

	Advanced Medical and Pharmacy Residents	First Year Wellness	Average Wellness in Females	Average Majority Pre‐COVID	Transitioning Through COVID	Doing Well
Pre‐COVID	0.267	0.292	0.294	**0.300**	0.259	0.233
Early COVID	0.185	0.236	0.215	0.260	**0.319**	0.256
COVID MID I	0.248	0.258	0.256	0.210	**0.301**	0.250
COVID MID II	**0.300**	0.213	0.236	0.229	0.120	0.261
PGY1	0.362	**0.389**	0.351	0.353	0.321	0.324
PGY2	**0.326**	0.265	0.264	0.279	0.278	0.223
PGY3	**0.270**	0.224	0.212	0.217	0.196	0.255
PGY4	0.041	0.074	0.112	0.094	0.**132**	0.086
PGY5	0.000	0.048	0.061	0.058	0.072	**0.112**
Male	0.530	0.571	0.468	0.535	0.632	**0.674**
Medical	**0.681**	0.676	0.622	0.638	0.647	0.620
Surgical	0.107	0.160	0.193	0.226	0.186	**0.243**
Pharmacy	**0.135**	0.054	0.048	0.033	0.058	0.011
Unspecified	0.077	0.110	**0.138**	0.104	0.109	0.126
Meaning	2.10	2.82	3.37	3.87	4.28	**4.90**
Human Connection	2.42	3.08	3.51	3.96	4.41	**4.85**
Overall	2.26	2.94	3.45	3.92	4.34	**4.89**

*Note:* Bolded values indicate the highest probabilities in each row. Underlined values indicate the lowest probabilities in each row.

Class 1, **Advanced Pharmacy and Medical Residents Wellness** (*n* = 48, 5%), was the smallest group and composed of individuals who had the lowest wellness scores. This class had the highest probability of having individuals who are in a medical programme (0.681) and pharmacy programme (0.135). This class also had the lowest probability of having been surveyed in early‐COVID‐19 (0.19) but had the highest probability of being surveyed in COVID‐19 Mid II (0.30). This group had the highest probabilities of individuals who were in their second and third years (0.33 and 0.27).

Class 2, **First Year Wellness** (*n* = 156, 15%), was characterised by having the second poorest scores on the wellness measure. This class had the second highest probability of individuals who were in a medical programme (0.676) but had the highest probability of individuals in their first year (0.389). This class was more likely to be male (0.571).

Class 3, **Average Wellness in Females**, was the second largest group (*n* = 278, 27%) and was characterised by the highest probability of being female (0.532). This class had the third lowest scores on the overall wellness measure and each subscale but had the highest probability of individuals who did not specify a programme type (0.138).

Class 4, **Average Majority Pre‐COVID** (*n* = 332, 32%), was characterised by being the largest class and having the third highest wellness scores. This class had the highest probability of being surveyed pre‐COVID‐19 (0.30) and had a higher probability of being male (0.535). Additionally, this class had the second highest probability of having surgical residents (0.236).

Class 5, **Transitioning Through COVID** (*n* = 131, 13%), was characterised by the lowest probability of individuals being surveyed during the last part of COVID‐19 (0.120) but had the highest probability of individuals being surveyed during the early part of COVID‐19 (0.319) and COVID‐19 Mid I (0.301). This class had the second highest scores on the wellness measure. Additionally, this class had the second highest probability of being male (0.632). Lastly, this class had the lowest probabilities of having PGY1 and PGY3 residents (0.321 and 0.196, respectively) but had the highest probability of having PGY4 residents (0.132).

Class 6, **Doing Well**, was the second smallest group (*n* = 88, 9%) and was characterised by having the highest scores on the wellness measure. This class had the highest probability of having individuals in a surgical programme (0.243) but had the lowest probability of individuals in a medical programme (0.620). Individuals in this class had the highest probability of being male (0.674). This class had the highest probability of having individuals in their fifth year (0.112).

## 4 Discussion

In this study, a LPA was utilised to identify whether residents' intersecting positionalities and timeframes throughout COVID‐19 impacted scores on wellness. Broadly, classes that had higher probabilities of being male, in surgical programmes, and were in Years 4 and 5 of their residencies had higher scores on the wellness measure. In terms of the influence of COVID‐19 on wellness scores, it was more difficult to ascertain a particular pattern. However, one class that had higher probabilities of residents being surveyed in 2022 had the lowest wellness scores.

### 4.1 Biological Sex

The two classes with the highest wellness scores had the highest probabilities of being male (i.e., Wellness Transition during COVID‐19 and Doing Well). The class with the highest probability of being female, Average Female Wellness, had the third lowest wellness scores, meaning that of the six classes, this class ranked in the bottom half in terms of wellness scores. These findings align with previous results that found that, generally, female residents had poorer subjective wellness ratings when compared to male residents [2, 3, 4]. This may indicate that the average female, regardless of residency type, year and COVID‐19 timeframe, have lower wellness scores than males. However, classes that were more mixed in terms of biological sex were more nuanced on their scores, which may be explained by residents' intersecting positionality.

### 4.2 Intersecting Identities

The two lowest scoring classes, Advanced Pharmacy and Medical Residents Wellness and First Year Wellness, were heterogenous in terms of biological sex but had the highest probabilities of individuals in a medical or pharmacy programme and who were early to midway through their training. This may indicate that medical and pharmacy residents early to midway through training tend to have lower scores on wellness measures when compared to surgical residents, particularly male surgical residents. Though intersectional research regarding residents is limited, Felton et al. found that surgery residents in PGY1 and PGY2 rated their wellness more poorly, which was further influenced by sex as male residents were more likely to perceive an increase in wellness when their PGY level changes [22]. Although this finding is specific to surgical residents, it aligns with theoretical implications—residents earlier in their training undoubtedly have less power and influence, which may be further exacerbated by being female. This may be especially true for frontline medical residents who experienced increased demands during COVID‐19.

### 4.3 COVID‐19

Broadly, we found it difficult to ascertain meaningful patterns based on COVID‐19 in several classes. Most classes' probabilities related to COVID‐19 time points ranged between 0.21 and 0.29. Whereas previous studies have found that COVID‐19 had an initial impact on residents' perceptions of wellness, other studies indicated that COVID‐19 did not contribute to meaningful changes pre‐ and post‐pandemic [9, 23, 24]. Findings from this study suggest reasons for this varying results—the pandemic may have uniquely impacted residents based on their intersecting identities.

The largest class, Average Majority Pre‐COVID‐19, most likely had the largest raw number of individuals surveyed pre‐COVID‐19 and therefore may represent what ‘average’ wellness looked like pre‐COVID‐19. However, the range of probabilities across time points was relatively narrow (0.21–0.30), which makes interpretation difficult. The Advanced Pharmacy and Medical Residents Wellness class had the lowest probability of being surveyed in early 2020 but had the highest probability of being surveyed in 2022. The Transitioning Through COVID‐19 class had the highest probabilities of being surveyed early in the pandemic but had the lowest probability of being surveyed in 2022. This may indicate that some residents (i.e., male and advanced surgical) had increased wellness throughout the first parts of COVID‐19, but a small subset (i.e., advanced pharmacy and medical) experienced a decrease in wellness during the later parts. These findings are partially aligned with previous results, which found that advanced medical students had increased mental health concerns during COVID‐19 [25]. However, our findings extend the research by indicating that programme type, not just year in training, directly influences wellness in periods of extended crisis, possibly due to increased stress of being on the frontlines. Indeed, previous research showed general decreases in sense of belonging in addition to significant differences in loneliness between surgical and medical residents during the latter part of 2020 [9, 26]. Further, other research found decreased wellness in students during the latter part of COVID‐19 than the first, particularly among females, which reinforces the temporal impact COVID‐19 may have had on certain groups of individuals [27].

### 4.4 Recommendations

Many resident wellness initiatives have been described in the literature, such as restrictions on duty hours, stress management treatments and mindfulness trainings, which are often initiated broadly across departments. However, our data suggest that wellness programming needs to be tailored by gender, programme type and year in programme, given that female, medical and pharmacy and early‐ to mid‐level residents consistently demonstrate more vulnerability. Broadly, these groups may benefit from wellness initiatives that focus on building social cohesion among and between cohorts (e.g., first years across all programmes), skills to navigate the power dynamics within the medical system and increased focus on diversity and equity considerations, despite political pressure to not do so. Given the political climate, fostering belongingness may be especially imperative for residents who are minorities and subsequently do not have as much power across systems of influence.

In this study, the Advanced Medical and Pharmacy Residents group may have benefited from continued emphasis on wellness that may have been present early during the pandemic, increased support as residents transitioned from PGY1 to PGY2, reminders of how their work aligned with their values and increased wellness initiatives across specialties (e.g., combining pharmacy and internal medicine residents) to increase belongingness and decrease loneliness. In addition to these suggestions, First Year Medical Residents may have benefited from increased support and understanding of policies to mitigate uncertainty and stress. The Average Female group may have benefited from increased mentorship and a thorough examination of institutional policy and resources to identify and mitigate gender bias. Regardless of these suggestions, future qualitative research should be used to elicit resident feedback to design more tailored wellness interventions.

### 4.5 Limitations

Unfortunately, we were unable to capture the experiences of people who are transgender or gender nonconforming. We were hesitant to code missing data as someone being a gender minority as some females may have been hesitant to share their sex if they were in a subspecialty or year that was male‐dominated and vice versa. Therefore, we were unable to accurately identify which missing response was due to missing data or actual non‐response. Future studies should include more expansive options for gender, race, sexual orientation and disabilities; however, programme directors and researchers should seriously weigh the unintended consequences of asking people to disclose certain identities. Studies from a single institution exponentially increase the potential for someone identifying a minority resident as being part of a study, which may impact safety. One way that researchers and programme directors could increase safety is by grouping data from across institutions, which will increase future studies' sample sizes while providing more safety for minority residents. Additionally, further study is necessary to better understand the construct validity of the RWS. Finally, these results are based on residents from a single institution in Appalachia, which may limit generalisability.

## 5 Conclusions

Gender, programme type and year in programme affect resident wellness, whereas COVID‐19 time point had a moderate impact on wellness.

## Author Contributions

**Sara Beachy:** software, methodology, formal analysis, visualisation, writing – original draft, writing – review and editing, conceptualisation. **Jessica Luzier:** conceptualisation, data curation, investigation, supervision, resources, project administration, writing – original draft, writing – review and editing. **Chantel Weisenmuller:** conceptualisation, writing – original draft, writing – review and editing, methodology. **Kathleen Bors:** conceptualisation, resources, project administration, investigation, writing – original draft, writing – review and editing. **Mary Ann Maurer:** conceptualisation, project administration, writing – original draft, writing – review and editing. **Amna Anees:** conceptualisation, investigation, writing – original draft, writing – review and editing, project administration. **Tiffany Lasky:** conceptualisation, writing – original draft, writing – review and editing. **Lisa Calderwood:** conceptualisation, project administration, writing – original draft, writing – review and editing.

## Conflicts of Interest

The authors declare no conflicts of interest.

## Data Availability

The data that support the findings of this study are available from Wayne State University. Restrictions apply to the availability of these data, which were used under license for this study. Data are available from the author(s) with the permission of Wayne State University.
